# Evaluation of the Radiation Dose–Volume Effects of Optic Nerves and Chiasm by Psychophysical, Electrophysiologic Tests, and Optical Coherence Tomography in Nasopharyngeal Carcinoma

**DOI:** 10.1177/1533034617711613

**Published:** 2017-06-06

**Authors:** Ozlem Ozkaya Akagunduz, Suzan Guven Yilmaz, Deniz Yalman, Berna Yuce, Elif Demirkilinc Biler, Filiz Afrashi, Mustafa Esassolak

**Affiliations:** 1 Ege University Faculty of Medicine, Department of Radiation Oncology, Izmir, Turkey; 2 Ege University Faculty of Medicine, Department of Ophthalmology, Izmir, Turkey; 3 Tepecik Education and Research Hospital, Izmir, Turkey

**Keywords:** radiation dose–volume effects, optic nerves and chiasm, radiation optic neuropathy, electrophysiologic tests, anterior visual pathways

## Abstract

**Purpose:**

To evaluate the radiation dose–volume effects of optic nerves and chiasm by visual psychophysical, electrophysiologic tests, and optical coherence tomography in patients with locally advanced nasopharyngeal carcinoma.

**Materials and Methods:**

A series of visual tests including visual acuity, visual field, contrast sensitivity, visual evoked potential, and optical coherence tomography were administered to 20 patients with locally advanced (T3-T4) nasopharyngeal carcinoma who were treated with definitive chemoradiotherapy. Volume that received 55 Gy (V_55_), mean dose (*D*_mean_), highest dose to 5% of the volume (D_5_), and maximum dose (D_max_) for optic nerves and chiasm were evaluated for each patient. Cutoff values were identified as V_55_: 50%, D_mean_: 50 Gy, D_5_: 55 Gy, and D_max_: 60 Gy. The effects of radiation dose–volume on ophthalmologic tests were evaluated.

**Results:**

Ophthalmological evaluation revealed optic neuropathy with simultaneous retinopathy in 6 eyes of 4 patients and radiation retinopathy alone in both eyes of 1 patient. Regarding radiation dose–volume effects of the optic nerve, significant detrimental effect of all parameters was observed on visual acuity. Visual field and contrast sensitivity were affected significantly with V_55_ ≥ 50% and D_mean_ ≥ 50 Gy. Visual evoked potential latency was affected significantly with D_mean_ ≥ 50 Gy, D_5_ ≥ 55 Gy, and D_max_ ≥ 60 Gy. For the chiasm, significant detrimental effect of all parameters was observed on visual acuity as well. Retinal nerve fiber layer thickness and visual evoked potential amplitude were not affected by any of the dose–volume parameters neither optic nerves nor chiasm.

**Conclusion:**

The volume receiving the threshold dose, mean dose, and 5% of the volume receiving the maximum dose are important parameters besides maximum dose to optic nerves and chiasm. A comprehensive ophthalmological evaluation including visual field, contrast sensitivity, visual evoked potential latency, and amplitude should be performed for these patients. Visual evoked potential latency is an objective predictor of vision loss before the onset of clinical signs.

## Introduction

Primary treatment of nasopharyngeal carcinoma is radiotherapy for early stage disease and chemoradiotherapy (CRT) for locally advanced stage. Due to the proximity of the nasopharynx to critical structures and the need for high radiation doses for local control, the risks of radiation-induced toxicities are substantial. Anterior visual pathways—optic nerve and chiasm—are especially under risk in locally advanced disease invading the base of the skull and extending to the cavernous or sphenoidal sinus or to the paraorbital regions.^1^ Although rare, injury of the anterior visual pathways results in serious complications due to optic neuropathy or retinopathy.^2^ Radiation-induced optic neuropathy (RION) is defined as a sudden, painless, irreversible visual loss in 1 or both eyes after a latency of months to years following irradiation. Although very rare, it severely affects patients’ quality of life. Vascular occlusion, demyelination, free radical injury, DNA damage, or blood–brain barrier damage are some of the proposed factors responsible for RION.^3^ Similarly, radiation retinopathy is defined as an occlusive vasculopathy secondary to retinal vascular endothelial cell damage that may cause ischemia. Both are reported to occur between 3 months and 8 years, with a peak at 1 to 1.5 years.^4,5^

Radiation Therapy Oncology Group/European Organization for Research and Treatment of Cancer late radiation morbidity criteria which defined the injury on anterior visual pathways focused only on visual acuity (VA), and the studies were mostly concentrated on loss of vision.^6–9^ Nevertheless, decrease in VA may be caused by many reasons such as dry eye, refractive error, cataract, and many other media opacities. Limiting the toxicity evaluation solely on VA may be insufficient and may cause difficulty in defining the relationship between VA, dose–volume threshold levels, and the severity of the complications. The vision may be affected subclinically without overt loss at or above the tolerance doses of the anterior visual pathways. Several studies investigated the subclinical damage to the anterior visual pathways and the relationship between radiation-associated factors such as total dose and fraction size in nasopharyngeal carcinoma.^10–14^ However, with technological advances in treatment delivery systems and treatment planning software, volumetric dose data also became very important. Studies evaluating the relationship between dose–volume levels and the subclinical damage using comprehensive visual tests are lacking.

**Figure 1.**
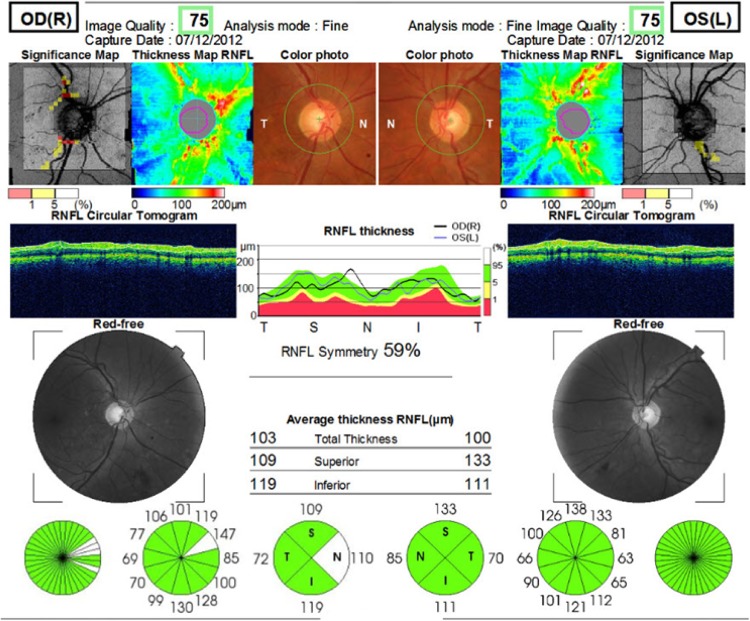
Typical optical coherence tomography (OCT) report (patient number 2, a 49-year-old male). The upper image illustrates the significance and thickness maps of retinal nerve fiber layer (RNFL) and color photos for the right oculus dexter (OD) and left oculus sinister (OS) eye, respectively. RNFL thickness is plotted with respect to a circumferential retinal map on the temporal-superior-nasal-inferior-temporal (TSNIT) quadrants (middle image). RNFL analysis demonstrating measurements within normative limits in both eyes, designated by a “stoplight” designation (bottom image). Note the quadrant and clockface sector measures of RNFL thickness.

Contrast sensitivity and visual field are important as much as VA for proper visual function. Visual field is the entire area that a person can see when staring at a fixed point. Visual field tests can determine the localization of the damage. Contrast sensitivity is the measurement of the minimum contrast required to distinguish a test object. It is a more sensitive way of measuring visual function. Low contrast sensitivity may affect a person’s daily life negatively despite a good VA. Visual evoked potential (VEP) is the physiologic response of the occipital cortex to a sensory stimulus of vision. Visual evoked potential latency is the transmission time of the visual stimulus from the optic nerve to the occipital cortex, whereas VEP amplitude is the height of this wave which defines the magnitude of the transmission. Visual evoked potentials can provide information about optic neuritis.^15^ Optical coherence tomography (OCT) creates high-resolution, cross-sectional microstructure images of the retina in real time.^16^ Retinal nerve fiber layer (RNFL) thickness surrounding the optic disc can be measured by OCT (Figure 1).

The purpose of the present study is to investigate the functions of the anterior visual pathways with psychophysical (VA, contrast sensitivity, and visual field), electrophysiologic (VEP latency and VEP amplitude) tests, and OCT in 20 patients with locally advanced nasopharyngeal carcinoma and to evaluate the relation between the results of the visual tests and radiation dose–volume effects of optic nerve and chiasm for each patient.

## Materials and Methods

### Patients and Treatment

The medical records of 20 patients with T3 and T4 nasopharyngeal carcinoma—according to 2010 American Joint Committee on Cancer Staging^17^—who were treated with definitive CRT at our institution between March 2007 and August 2012 were reviewed retrospectively after institutional review board approval. Patients with recurrent disease, history of prior radiotherapy to the same region, radiotherapy portals not including the optic nerve and chiasm, tumor invasion to the optic nerve and chiasm, vision loss, advanced refractive error, glaucoma, or keratitis history prior to radiotherapy and noncompliant patients were excluded. Median age was 49 (range: 18-65) years. Sixteen (80%) patients were males. Twelve patients (60%) had T4 tumor.

Three dimensional conformal radiotherapy was planned for 18 (90%) patients and volumetric arc therapy for 2 patients using 6 MV X-rays. Total radiotherapy dose to the gross tumor volume was 70 Gy with 2 to 2.12 Gy daily fractions, 59 to 61 Gy to high-risk area, and 50 to 54 Gy to low-risk area. The prescription dose is the isodose surface that encompasses 95% of the planning target volume (PTV). No more than 20% of any PTV_70_ received ≥110% of the prescribed dose. No more than %1 of any PTV_70_ received ≤93% of the prescribed dose. The patients received 3 cycles of induction chemotherapy with cisplatin (75 mg/m^2^) and docetaxel (75 mg/m^2^) followed by cisplatin (75 mg/m^2^) concomitant with RT, at 3-week intervals.

Volume that received 55 Gy (V_55_), mean dose (D_mean_), highest dose to 5% of the volume (D_5_), and maximum dose (D_max_) for right and left optic nerves and chiasm were evaluated according to the dose–volume histograms of each patient. Cutoff values were identified as V_55_: 50%, D_mean_: 50 Gy, D_5_: 55 Gy, and D_max_: 60 Gy. The correlation between the results obtained from the ophthalmologic tests and the values below and above the cutoff was evaluated.

### Ophthalmological Evaluation

Visual tests were performed at a median of 41 (range: 12-60) months after the completion of CRT. Visual acuity, anterior and posterior segment evaluations followed by visual field, contrast sensitivity, and VEP tests were done; and RNFL thickness measurements obtained by OCT were recorded for 40 eyes of 20 patients.

Visual acuity was measured with Snellen chart on the basis of the autorefractometer results after refractive correction. Visual field was analyzed via Zeiss Humphrey Field Analyzer (Carl Zeiss Meditec AG, Berlin, Germany) using a 120 full-field protocol after correction of near-vision correlated with age if needed. Visual field defects were scored as grade 1: normal; grade 2: peripheral defects outside the central 30° field; grade 3: defects located in the central 30° field; grade 4: generalized defects.

Contrast sensitivity was measured by MetroVision (Monopack 3, Perenchies, France) under photopic conditions designating contrast sensitivity between 0.5 and 15 cycle/degree spatial frequencies. The contrast sensitivity value corresponding to each spatial frequency was plotted, and the attained line was compared to the normal line. It was scored as Grade 1: normal; Grade 2: under the normal line beginning from high-spatial frequencies (5 cycle/degree); Grade 3: under the normal line beginning from low spatial frequencies (1 cycle/degree) for each eye.

Visual evoked potentials were obtained via MetroVision using pattern reversal stimulus. The latency and amplitude of the P100 potentials for each eye was recorded in milliseconds and microvolts, respectively. The reference range of VEP latency and amplitude can differ from one center to another according to the equipment used and environmental factors. In the present study, the normal range of VEP latency for the P100 potential was 106 ± 3 milliseconds, and the VEP amplitude for the P100 potential was 9 ± 4 µV, according to the normal population measurements made previously using the equipment mentioned above. Amplitudes lower than normal and/or delay in latency are considered pathological.

Retinal nerve fiber layer measurements were done by spectral domain OCT (Topcon RM-8000B, Topcon Corporation, Tokyo, Japan) following pupillary dilatation. The values between 90 and 110 µm were considered normal.

### Statistical Analysis

PASW Statistics version 18 software was used for the statistical analyses. Significance level of α, for type I error, was accepted as 0.05. Relationship between upper and lower cutoff values of radiation dose–volume parameters and ophthalmologic tests were assessed using Pearson χ^2^.

## Results

Forty eyes of 20 patients were evaluated. Ophthalmological evaluation revealed optic neuropathy with simultaneous retinopathy in 6 eyes of 4 patients and radiation retinopathy alone in both eyes of 1 patient. Involvement was bilateral in 3 patients and unilateral in 2 patients. Retinal exudates and hemorrhage related to radiation retinopathy, optic disc edema, and splinter hemorrhages related to optic neuropathy were seen on fundus examination. Visual acuity was decreased in 6 eyes of 4 patients with optic neuropathy and radiation retinopathy. Visual acuity was not affected (VA: 10/10) in the left eye of patient 2 with isolated radiation retinopathy because of the lack of macula involvement, and also VEP latency was normal in this eye because there was no optic neuropathy accompanying retinopathy. Radiation retinopathy occurred 30 months after radiotherapy in patient 10. When his previous clinical chart which was recorded 1 year ago was reevaluated, it was seen that VA was 10/10 for both eyes despite significant delay in VEP latency at that time (right eye: 143 milliseconds and left eye: 137 milliseconds). The ophthalmological evaluation results of the whole group were indicated in Table 1.

**Table 1.** Ophthalmological Evaluation Results of the Patients.

Patient No.	Laterality	Visual Acuity	Visual Field	Contrast Sensitivity	VEP Latency, milliseconds	VEP Amplitude, µV	RNFL Thickness	Optic Neuropathy With or Without Retinopathy
1	Left eye	1.0	2	1	107	5.6	94	No
	Right eye	1.0	1	1	109	8.5	94	No
2	Left eye	0.4	4	3	103	3.5	103	Yes
	Right eye	1.0	2	2	105	4.6	100	Yes
3	Left eye	0.1	3	3	111	9.2	98	No
	Right eye	0.05	4	3	114	8.3	99	No
4	Left eye	1.0	1	1	103	16.1	110	No
	Right eye	1.0	1	1	106	14.4	122	No
5	Left eye	1.0	1	1	94	8.5	99	No
	Right eye	1.0	3	1	95	6.7	96	No
6	Left eye	0.7	3	1	94	6.4	96	No
	Right eye	1.0	2	1	95	3.3	92	No
7	Left eye	1.0	2	1	98	9.9	99	No
	Right eye	1.0	2	1	98	12.1	51	No
8	Left eye	1.0	2	1	107	9.9	97	No
	Right eye	0.9	2	1	108	8.1	94	No
9	Left eye	0.9	2	1	108	2.3	92	No
	Right eye	1.0	2	1	109	3.6	98	No
10	Left eye	1.0	4	3	143	10.4	94	Yes
	Right eye	0.1	4	2	148	5.2	91	Yes
11	Left eye	1.0	1	1	109	11.1	92	No
	Right eye	1.0	1	1	103	11.1	78	No
12	Left eye	1.0	3	3	114	6.9	90	No
	Right eye	1.0	4	3	112	4.4	77	No
13	Left eye	1.0	2	3	109	2.9	92	No
	Right eye	1.0	2	3	106	5.6	92	No
14	Left eye	0.9	3	2	95	2.1	83	No
	Right eye	0.9	3	2	108	3.2	95	No
15	Left eye	0.4	1	1	101	5.2	85	No
	Right eye	0.03	4	3	139	4.3	85	Yes
16	Left eye	1.0	2	1	98	12.3	51	No
	Right eye	1.0	2	1	97	9.9	99	No
17	Left eye	0.3	4	3	127	2.2	93	Yes
	Right eye	0.6	4	3	127	4.3	95	Yes
18	Left eye	1.0	2	1	111	4.1	96	No
	Right eye	0.08	Unable	Unable	156	1.2	96	Yes
19	Left eye	1.0	4	3	98	16.1	94	No
	Right eye	1.0	4	3	103	1.5	95	No
20	Left eye	1.0	2	2	105	8.3	81	No
	Right eye	0.9	3	2	99	7.6	99	No

Abbreviations: RNFL, retinal nerve fiber layer; VEP, visual evoked potential.

**Figure 2.**
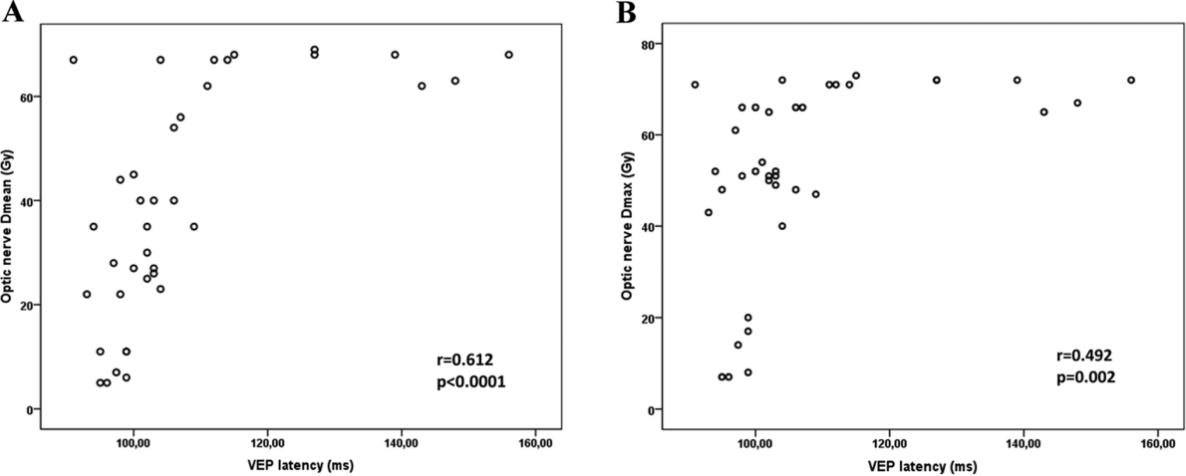
Correlation between D_mean_ (A), D_max_ (B), and visual evoked potential (VEP) latency for optic nerves.

Regarding radiation dose–volume effects of the optic nerves, significant detrimental effect of all parameters (V_55_ ≥ 50%, D_mean_ ≥ 50 Gy, D_5_ ≥ 55 Gy, and D_max_ ≥ 60 Gy) was observed on VA (*P* = .002, *P* = .001, *P* = .008, and *P* = .008, respectively). Visual field and contrast sensitivity were affected both with V_55_ ≥ 50% and D_mean_ ≥ 50 Gy (*P* = .045, *P* = .050, *P* = .024, and *P* = .003). Visual evoked potential latency prolonged significantly with D_mean_ ≥ 50 Gy (*P* < .001), D_5_ ≥ 55 Gy (*P* = .005), and D_max_ ≥ 60 Gy (*P* = .002). Figure 2 shows the correlation between D_mean_, D_max_, and VEP latency for the optic nerve. Retinal nerve fiber layer thickness and VEP amplitude were the only ophthalmologic parameters that were not affected by any of the dose–volume parameters (Table 2).

**Table 2.** The Effect of Radiation Dose–Volume Parameters on Ophthalmologic Tests for Optic Nerves.^a^

Visual Tests	Median Score	Odds Ratio	Confidence Interval (95% CI)	*P* Value
**Visual acuity**				
V_55_ <50% vs ≥50%	1.0 vs 0.6	18.85	1.92-184.63	.002
D_mean_ <50 Gy vs ≥50 Gy	1.0 vs 0.4	28.80	2.81-188.80	.001
D_5_ <55 Gy vs ≥55 Gy	1.0 vs 0.7	13.33	1.39-127.57	.008
D_max_ <60 Gy vs ≥60 Gy	1.0 vs 0.7	13.33	1.39-127.57	.008
**RNFL thickness**				
D_mean_ <50 Gy vs ≥50 Gy	94 vs 95	1.03	0.21-5.01	.965
D_max_ <60 Gy vs ≥60 Gy	94 vs 95	0.62	0.14-2.71	.529
**Visual field**				
V_55_ <50% vs ≥50%	2 vs 3	4.23	1.45-39.87	.045
D_mean_ <50 Gy vs ≥50 Gy	2 vs 3	0.72	0.56-0.91	.050
D_5_ <55 Gy vs ≥55 Gy	2 vs 3	5.60	0.59-52.53	.102
D_max_ <60 Gy vs ≥60 Gy	2 vs 3	5.60	0.59-52.53	.102
**Contrast sensitivity**				
V_55_ <50% vs ≥50%	1 vs 3	5.14	1.17-22.48	.024
D_mean_ <50 Gy vs ≥50 Gy	1 vs 3	11.57	1.98-67.49	.003
D_5_ <55 Gy vs ≥55 Gy	1 vs 3	5.00	0.19-20.92	.123
D_max_ <60 Gy vs ≥60 Gy	1 vs 3	5.00	0.19-20.92	.123
**VEP latency**				
V_55_ <50% vs ≥50%	101 vs 110	2.48	0.61-10.05	.096
D_mean_ <50 Gy vs ≥50 Gy	100 vs 117	26.62	4.52-155.34	<.001
D_5_ <55 Gy vs ≥55 Gy	100 vs 113	7.71	1.71-36.63	.005
D_max_ <60 Gy vs ≥60 Gy	99 vs 112	9.52	2.01-44.91	.002
**VEP amplitude**				
V_55_ <50% vs ≥50%	8.4 vs 5.3	1.60	0.34-7.45	.54
D_mean_ <50 Gy vs ≥50 Gy	8.5 vs 4.8	1.18	0.23-5.96	.83
D_5_ <55 Gy vs ≥55 Gy	8.4 vs 6.0	1.16	0.25-5.33	.84
D_max_ <60 Gy vs ≥60 Gy	8.4 vs 6.2	2.12	0.46-9.80	.32

Abbreviations: CI, confidence interval; OR, odds ratio; RNFL, retinal nerve fiber layer; VEP, visual evoked potential.

^a^Visual acuity: 1.0: normal, <1.0: abnormal; Visual field: Grade 2: peripheral defects outside the central 30° field, grade 3, defects located in the central 30° field; contrast sensitivity: grade 1: normal, grade 3: under the normal line beginning from low spatial frequencies (1 cycle/degree) for each eye. VEP latency: 106 ± 3 milliseconds; VEP amplitude: 9 ± 4 µV; odds ratio: cross tabulations were prepared, and odds ratios were calculated using the formula, OR = a × d/b × c.

**Figure 3.**
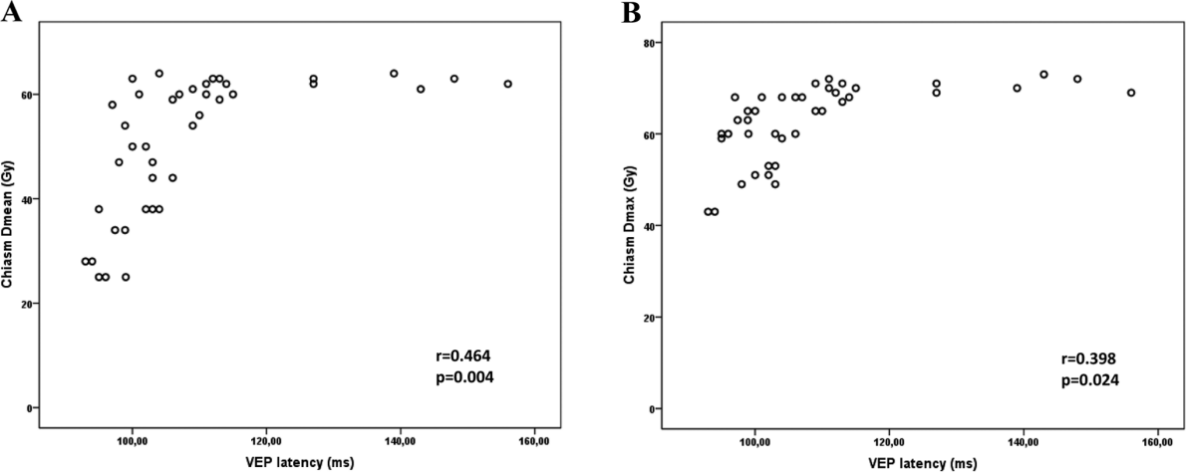
Correlation between D_mean_ (A), Dmax (B), and visual evoked potential (VEP) latency for chiasm.

In terms of radiation dose–volume effects of the chiasm, significant detrimental effect of all parameters (V_55_ ≥ 50%, D_mean_ ≥ 50 Gy, D_5_ ≥ 55 Gy, and D_max_ ≥ 60 Gy) was also observed on VA (*P* = .008, *P* = .003, *P* = .037, and *P* = .019, respectively). D_mean_ ≥ 50 Gy significantly affected visual field (*P* = .035), contrast sensitivity (*P* = .007), and VEP latency (*P* = .005). D_max_ ≥ 60 Gy had a detrimental effect on contrast sensitivity and VEP latency as well (*P* = .023 and *P* = .045, respectively). Figure 3 shows the correlation between D_mean_, D_max_, and VEP latency for the chiasm. Once more, RNFL thickness and VEP amplitude were the only ophthalmologic parameters that were not affected by any of the dose–volume parameters for the chiasm (Table 3).

**Table 3.** The Effect of Radiation Dose–Volume Parameters on Ophthalmologic Tests for Chiasm.^a^

Visual Tests	Median Score	Odds Ratio	Confidence Interval (95% CI)	*P* Value
**Visual acuity**				
V_55_ <50% vs ≥50%	1.0 vs 0.3	13.33	1.39-127.57	.008
D_mean_ <50 Gy vs ≥50 Gy	1.0 vs 0.7	1.63	1.13-2.36	.003
D_5_ <55 Gy vs ≥55 Gy	1.0 vs 0.7	1.41	1.09-1.82	.037
D_max_ <60 Gy vs ≥60 Gy	1.0 vs 0.8	1.46	1.10-1.95	.019
**RNFL thickness**				
D_mean_ <50 Gy vs ≥50 Gy	90 vs 93	1.00	0.23-4.30	1.00
D_max_ <60 Gy vs ≥60 Gy	94 vs 89	0.58	0.12-2.78	.49
**Visual field**				
V_55_ <50% vs ≥50%	2 vs 3	4.23	4.50-39.87	.180
D_mean_ <50 Gy vs ≥50 Gy	2 vs 3	8.51	0.90-80.02	.035
D_5_ <55 Gy vs ≥55 Gy	2 vs 3	3.50	0.63-19.23	.137
D_max_ <60 Gy vs ≥60 Gy	2 vs 3	5.62	0.44-22.53	.102
**Contrast sensitivity**				
V_55_ <50% vs ≥50%	1 vs 3	3.00	0.75-11.86	.112
D_mean_ <50 Gy vs ≥50 Gy	1 vs 3	7.00	1.59-30.80	.007
D_5_ <55 Gy vs ≥55 Gy	1 vs 3	2.00	0.47-8.46	.343
D_max_ <60 Gy vs ≥60 Gy	1 vs 3	5.00	1.19-20.92	.023
**VEP latency**				
V_55_ <50% vs ≥50%	101 vs 113	7.91	1.71-36.63	.005
D_mean_ <50 Gy vs ≥50 Gy	97 vs 110	9.35	1.71-51.03	.005
D_5_ <55 Gy vs ≥55 Gy	99 vs 110	3.78	0.69-20.51	.109
D_max_ <60 Gy vs ≥60 Gy	99 vs 107	5.10	0.94-27.54	.045
**VEP amplitude**				
V_55_ <50% vs ≥50%	8.5 vs 5.0	1.16	0.25-5.33	.845
D_mean_ <50 Gy vs ≥50 Gy	9.3 vs 5.2	2.5	0.51-12.13	.248
D_5_ <55 Gy vs ≥55 Gy	7.6 vs 5.6	1.0	0.20-4.95	1.00
D_max_ <60 Gy vs ≥60 Gy	8.9 vs 6.7	1.37	0.28-6.70	.693

Abbreviations: CI, confidence interval; OR, odds ratio; RNFL, retinal nerve fiber layer; VEP, visual evoked potential.

^a^ Visual acuity: 1.0: normal, <1.0: abnormal; visual field: grade 2: peripheral defects outside the central 30° field, grade 3: defects located in the central 30° field; contrast sensitivity: grade 1: normal, grade 3: under the normal line beginning from low spatial frequencies (1 cycle/degree) for each eye. VEP latency: 106 ± 3 milliseconds; VEP amplitude: 9 ± 4 µV; odds ratio: cross tabulations were prepared, and odds ratios were calculated using the formula, OR = a × d/b × c.

## Discussion

Most of the previous studies evaluating radiation damage to the anterior visual pathways are focused only on VA. Nevertheless, it must be kept in mind that radiation damage to the anterior visual pathways may occur before the development of VA loss or without any loss in VA. Many studies demonstrated that deterioration in contrast sensitivity, visual field, and RNFL thickness may cause difficulty in a patient’s daily life.^18–23^ So, in the present study, radiation dose–volume damage relation on anterior visual pathways was evaluated by visual field, contrast sensitivity, RNFL thickness, VEP latency, and VEP amplitude measurements besides VA.

In general, studies evaluating the radiation damage by VA tried to determine a maximum threshold dose for optic nerves and chiasm. Parsons *et al* reported that none of the patients in their study developed RION with a D_max_ < 59 Gy.^7^ Jiang *et al* stated that radiation dose <56 Gy did not cause RION, and the incidence is <5% at 10 years for a dose <60 Gy at ≤2.5 Gy/fraction.^14^ Martel and associates notified that D_max_ ≥64 Gy with V_60_ >25% for optic nerves may cause moderate to severe complications on visual pathways.^8^ In the present study, V_55_ ≥50%, D_mean_ ≥50 Gy, D_5_ ≥55 Gy, and D_max_ ≥60 Gy for the optic nerves caused a significant decrease in VA (*P* = .002, *P* = .001, *P* = .008, and *P* = .008, respectively). Radiation-induced optic neuropathy accompanying radiation retinopathy was detected in 4 patients and radiation retinopathy without RION occurred in 1 patient who received higher doses. It was observed that a decrease in VA was accompanied by a deterioration in visual field and contrast sensitivity in our patients if V_55_ ≥50% (*P* = .045 and *P* = .024, respectively), and D_mean_ ≥50 Gy (*P* = .050 and *P* = .003) for the optic nerves.

It is obvious that subjective tests evaluating the clinical evidence of damage on anterior visual pathways can be highly affected by the intellectual capacity and the compliance of the patient, so objective tests are crucial. Electrophysiologic tests (VEP latency and VEP amplitude) have been found to be more objective and sensitive in detecting occult disorders. These tests may be abnormal months before the loss of vision in patients with anterior visual pathway radionecrosis.^12,24,25^ Esassolak *et al* investigated the functions of the anterior visual pathways in locally advanced nasopharyngeal carcinoma patients both by electrophysiologic and psychophysical (VA and contrast sensitivity) tests and found that the radiation damage to the optic nerves and chiasm did not show statistically significant differences for doses over 50 Gy, but VEP latency, VEP amplitude, contrast sensitivity, and visual field were affected negatively in the radiotherapy group when compared with the control group.^10^ Pan *et al* investigated optic neuropathy in 28 patients with nasopharyngeal carcinoma by visual field and VEP tests before irradiation, at the end, and 5 years after irradiation.^11^ They found significant delay in VEP latencies and nonsignificant decrease in VEP amplitudes within 1 year after irradiation than preirradiation in 78.6% of the investigated eyes. After 1 year, decrease in VEP amplitudes also became significant.

Hasegawa and associates analyzed the tolerance dose for retention of VA in 30 patients with head-and-neck tumors treated with carbon ion radiotherapy.^26^ They performed VA tests, fundoscopy, visual field tests, and VEP tests, and found a correlation between the occurrence of visual loss and a delivery of >60 GyE to 20% of the volume of the optic nerve. In the present study, D_mean_ ≥50 Gy, D_5_ ≥55 Gy, and D_max_ ≥60 Gy caused delay in VEP latency for the optic nerves (*P* < .001, *P* = .005, and *P* = .002, respectively). The prolongation of VEP latency even in case of a maximum point dose above the threshold value (D_max_ ≥60 Gy) gave an impression that VEP latency may be affected without any clinical sign. In their study which assessed the dose–response tolerance of the visual pathways and cranial nerves of the cavernous sinus to stereotactic radiosurgery, Leber *et al* stated that VEP may be abnormal months before the loss of vision in patients with anterior visual pathway radionecrosis.^25^ In our study, one of the patients who developed RION 30 months after radiotherapy had previous records of ophthalmologic tests which were done 1 year ago, and in his previous clinical chart VA was 10/10 for both eyes despite significant delay in VEP latency (right eye: 143 milliseconds and left eye: 137 milliseconds). Visual evoked potential latency might provide early evidence of optic neuropathy before the onset of visual symptoms. It is obvious that VA alone is insufficient when evaluating the effects of radiotherapy on anterior visual pathways.

Ophthalmological evaluation revealed radiation retinopathy either alone or accompanying RION in 5 patients although retina was not included in the radiotherapy field. As mentioned before, radiation retinopathy is defined as an occlusive vasculopathy secondary to retinal vascular endothelial cell damage after radiotherapy that may cause ischemia.^5^ Microangiopathic changes similar to those in diabetes, venous occlusive diseases, and telangiectatic disorders may occur in retina after radiotherapy.^27^ Even though radiotherapy does not affect retina directly, it may cause retrograde degeneration in retinal ganglion cells via the optic nerve damage or microvascular damage which predisposes to retinopathy.^28,29^

Previous studies mostly used VA, visual field, contrast sensitivity, and VEP tests. Use of OCT is rare. In the present study, RNFL thickness which was determined by OCT is the sole parameter which was not affected by radiation. It may be based on the fact that the impact of radiation occurs on the optic nerve pathway in the radiotherapy field, not primarily on retinal ganglion cell axons and neurons as it is in inflammatory diseases such as multiple sclerosis.^21^

The limitations of the present study are the relatively small number of patients evaluated, lack of preradiotherapy, and regular ophthalmological evaluations after radiotherapy as well as its retrospective nature. However, to the best of our knowledge, this is the first study evaluating the relationship between dose–volume parameters of the anterior visual pathway and visual function by more comprehensive ophthalmologic tests. A larger prospective study is required to achieve more reliable results.

## Conclusions

Despite their rarity, RION and radiation retinopathy are the important complications that may occur in patients treated with radiotherapy for nasopharyngeal carcinoma. The disabling damage to vision severely affects the patient’s quality of life, so a careful follow-up is crucial besides a careful radiotherapy planning. Previous studies have evaluated the maximum dose received by the optic nerve and chiasm. Volume dependence is not well understood. Nevertheless, in the modern radiotherapy era, the volume of the critical organ receiving a particular dose is very important. Besides, during daily treatment process, it is quite difficult to obtain the maximum point dose at the same point each and every day. For this reason, the volume receiving the threshold dose, mean dose received by the critical structure, and 5% of the volume receiving the maximum dose are important parameters to be evaluated.

Regarding the visual assessment in these patients, VA test alone is inadequate due to its subjective nature and lack of reflecting the severity of subclinical damage. A more comprehensive ophthalmological evaluation including visual field, contrast sensitivity, VEP latency, and amplitude should be performed. Especially electrophysiological tests help in the early detection of radiation damage to the visual pathway. Visual evoked potential latency is quite important as an objective predictor of vision loss before the onset of clinical signs. Early recognition of RION and treatment in incipient phases is very important. No treatment has been proven effective, visual prognosis is poor, so one must be aware of this side effect. The damage caused by the dose–volume effects of irradiation on anterior visual pathways at clinical and subclinical levels will gain importance with the radiobiological results of new radiotherapy techniques, different fractionation schemes, and fraction doses. Studies regarding the potential medical or interventional treatment of radiation-induced damage to the visual pathway are needed.

## Abbreviations

CRT: chemoradiotherapy
OCT: optical coherence tomography
PTV: planning target volume
RION: radiation-induced optic neuropathy
RNFL: retinal nerve fiber layer
VA: visual acuity
VEP: visual evoked potential
